# Effects of virtual reality training on occupational performance and self-efficacy of patients with stroke: a randomized controlled trial

**DOI:** 10.1186/s12984-020-00783-2

**Published:** 2020-11-13

**Authors:** Yi Long, Rang-ge Ouyang, Jia-qi Zhang

**Affiliations:** 1 Department of Rehabilitation, Xiangya Hospital Central South University, No. 87 Xiangya Road, Changsha, 410008 Hunan China; 2 Department of Rehabilitation Sciences, The Hong Kong Polytechnic University, Hong Kong SAR, China

**Keywords:** Virtual reality, Occupational performance, Self-efficacy, Stroke, Rehabilitation

## Abstract

**Background:**

Virtual reality (VR) has been broadly applied in post-stroke rehabilitation. However, studies on occupational performance and self-efficacy as primary outcomes of stroke rehabilitation using VR are lacking. Thus, this study aims to investigate the effects of VR training on occupational performance and self-efficacy in patients with stroke.

**Methods:**

This was an assessor-blinded, randomized controlled trial. Sixty participants with first-ever stroke (< 1-year onset) underwent rehabilitation in a single acute hospital. Participants were randomly assigned to either the VR group (n = 30) or control group (n = 30). Both groups received dose-matched conventional rehabilitation (i.e., 45 min, five times per week over 3 weeks). The VR group received additional 45-min VR training for five weekdays over 3 weeks. The primary outcome measures were the Canadian Occupational Performance Measure and Stroke Self-Efficacy Questionnaire. Secondary outcome measures included Modified Barthel Index, Fugl-Meyer Assessment-Upper Extremity, and Functional Test for the Hemiplegic Upper Extremity. The assessment was conducted at baseline and after the 3-week intervention.

**Results:**

A total of 52 participants (86.7%) completed the trial. Significant between-group differences in Stroke Self-Efficacy Questionnaire (Median Difference = 8, *P* = 0.043) and Modified Barthel Index (Median Difference = 10, *P* = 0.030) were found; however, no significant between-group differences in Canadian Occupational Performance Measure, Fugl-Meyer Assessment-Upper Extremity, and Functional Test for the Hemiplegic Upper Extremity were noted. No serious adverse reactions related to the program were reported.

**Conclusions:**

Additional VR training could help improve the self-efficacy and activities of daily living of patients with stroke; however, it was not superior to conventional training in the improvement of upper limb functions, occupational performance, and satisfaction. Nevertheless, VR could be integrated into conventional rehabilitation programs to enhance self-efficacy of patients after stroke.

**Trial registration:**

This study was successfully registered under the title “Effects of virtual reality training on occupational performance and self-efficacy of patients with stroke” on October 13 2019 and could be located in https://www.chictr.org with the study identifier ChiCTR1900026550.

## Background

Stroke is a common neurological disease associated with high morbidity, mortality, and disability rates [1]. The survival rate of this disease has significantly improved in recent years. However, there are still a large number of stroke survivors experiencing severe dysfunctions. For instance, 85% of stroke survivors had upper extremity dysfunction at the initial stage of onset [2], and approximately 30–36% of patients with stroke still had upper extremity dysfunction at 6 months after stroke [3]. Such dysfunctions could greatly affect patients’ quality of life and prognosis after stroke, and improvement in the ability to complete activities of daily living (ADL) is vital for functional recovery.

Apart from sensation, motor function, and ADL, some important domains, including occupational performance and self-efficacy, have to be considered in stroke rehabilitation [4]. Occupational performance is the ability of the patients to handle different activities in the areas of self-maintenance, productivity, and leisure. Patients with good occupational performance could better perceive, desire, recall, plan, and perform roles, routines, and tasks to adapt to the internal or/and external environment [5]. Self-efficacy is defined as a person's ability to perform a task or activity with confidence [6]. Stroke Self-Efficacy Questionnaire (SSEQ) is the most commonly used measure for self-efficacy in patients with stroke. Compared with other self-efficacy scales for a specific dysfunction or tasks, such as the Self-Efficacy for Exercise Scale and the Falls Efficacy Scale, SSEQ covers all functional tasks and self-management items related to holistic self-efficacy for stroke population [7]. Four principles could enhance self-efficacy, which include direct mastery of experience, substitution of experience, verbal persuasion, and understanding of physiological states and signals [8]. A previous study found that self-efficacy is a moderator of occupational performance in stroke self-management programs [9].

Virtual reality (VR) equipment has become a popular and common rehabilitation tool in numerous hospitals [10]. VR is a computer technology that enables interaction between users and the environment and provides high doses of repetitive task training [11, 12]. The training provides an additional option for the participants, which in turn enhances their motivation of getting involved with daily training. Optimal use of VR equipment could save labor costs in acute hospitals. Previous reviews have suggested that VR is a promising, safe, novel, and exciting treatment tool [13–15], especially for the improvement of upper limb function, balance function, and gait [16–19]. However, in recently published reviews, no significant difference in the improvement of upper limb function between VR training and conventional training was found. VR training could improve the ADL of patients with stroke, the evidence on improved quality of life and participation restriction is limited [20–22]. Some studies showed that different outcome measures related to the effect of VR training still need to be specified [23–25]. The number of published clinical trials that used occupational performance [26, 27] and self-efficacy [22, 28] as primary outcomes in VR training are few.

In clinical practice, VR training may provide guidance and timely feedback to participants via simulated daily life virtual scene and demonstrations [12], which differs from conventional rehabilitation; the ability acquired in VR training may be better generalized to daily activities. Moreover, VR training could provide participants feedback on their body, voice, and vision as well as timely encouragement and support from therapists and family members [29]. Thus, it may be easier for the participants to understand their own performance and improve their sense of achievement [30], which could in turn improve enthusiasm and confidence in daily activities. Such characteristics of VR interventions are related to the four principles that could improve self-efficacy. Hence, our study will further explore the effect of VR training on self-efficacy and occupational performance.

Currently, a few VR-related studies confirmed a positive effect on occupational performance among patients with stroke [27, 31]. A few studies on VR combined with treadmills found significant improvements in falls-related self-efficacy and balance self-efficacy [22, 32, 33]. However, the evidence on the effect of VR on holistic self-efficacy of patients with stroke is lacking. Thus, VR-related studies that focus on the improvement of occupational performance and self-efficacy of patients with stroke are meaningful. Moreover, improvement of upper limb function by VR training also needs to be investigated.

Our study, which was designed as a randomized controlled trial, aimed to investigate the effect of VR on self-efficacy and occupational performance of patients with stroke. We hypothesized that VR plus conventional training were superior to conventional training in the improvement of self-efficacy, occupational performance and ADL. The results of upper limb function were equivalent in both groups.

## Methods

### Study design and participants

This study was an assessor-blinded randomized controlled trial and was reported according to the CONSORT statement for non-pharmacological trials. Participants were recruited consecutively at a large acute hospital in Changsha, China, from October 2019 to March 2020. Both outpatients and inpatients were included. Inclusion criteria were as follows: (1) diagnosis of first-ever stroke (onset time ≤ 1 year); (2) ability to follow verbal instructions (Mini-Mental State Evaluation score ≥ 24 points); an adjusted cut-off value of 17 was used for participants with formal education years < 6 [34]; (3) the muscle tone of upper limb evaluated by modified Ashworth scale < 2; (4) proximal upper limb strength assessed by manual muscle test ≥ 2; and (5) no visual field deficit or hemianopia. Participants with bilateral hemispheric stroke and severe cardiopulmonary diseases or other medical diseases that could affect their capacity to do rehabilitative training were excluded. Written informed consent was obtained from each participant prior to data collection. The participants were randomly assigned to two groups: VR (n = 30) or control (n = 30) groups.

### Intervention

The equipment used in this study was Doctor Kinetic (DIH Medical, Fengtai District, Beijing, China). The main component of the VR-based game system was a touch-controlled computer screen, which was used to input basic information, assess participants’ range of motion, and set personalized prescriptions. Infrared sensor smart recognition camera was used to track the range of joint movement, mainly that of large joints (shoulder and elbow). The camera sensor could capture the movements of the participant at a distance between 1.5 and 3.0 m. A human-shaped model on the computer screen demonstrated the required joint movements before the training, as well as some game actions. Participants must follow the actions of the human-shaped model to complete tasks one by one.

Participants in the VR group received VR training for 45 min, five times per week over 3 weeks. Before training, each participant was evaluated for active range of motion, including flexion, extension, adduction and abduction of shoulder joint. The VR games contained five tasks: bilateral upper limb flexion; abduction activity (20 times per activity); gold coins picking game, including shoulder circle; cross and mixed training for 3–5 min. The training difficulty and intensity were adjusted according to the participant's ability. To prevent falls, therapists stood behind the participant for safety and guidance, and the participant was allowed to grab the handrail, if necessary. During the training process, the participants’ feelings were also considered to avoid fatigue and overload training. Both groups received the same dosage of conventional training, which included occupational therapy, physical therapy, and acupuncture.

### Outcome measures

All outcomes were evaluated before and immediately after the 3-week intervention. The primary outcomes were occupational performance and self-efficacy, which were assessed by Canadian Occupational Performance Measure (COPM) and Stroke Self-Efficacy Questionnaire (SSEQ), respectively. COPM is a reliable and valid tool that measures self-perception of occupational performance in the areas of self-maintenance, leisure, and productivity. A change score of 2 was considered clinically significant, according to a previous report [35]. SSEQ was used to assess self-efficacy. It consists of two parts with 13 subitems, including daily activities and self-management.

The secondary outcomes were upper limb functions and ADL. Fugl-Meyer Assessment-Upper Extremity (FMA-UE) and Functional Test for the Hemiplegic Upper Extremity (FTHUE) were used to assess the upper limb functions. FMA-UE evaluates the motor function of the upper limbs, wrists, and hands. Change score of upper extremity ranged from 4.25 to 7.25 was considered to be clinically significant in stroke patients with minimal to moderate hemiparesis [36]. FTHUE is commonly used to evaluate the overall function of the upper extremity after stroke, consists of 13 domains, and has seven functional levels. Modified Barthel Index (MBI), which used a five-step scoring system, was used to assess ADL.

### Sample size

The sample size was calculated based on published studies. The effect size of COPM performance, COPM satisfaction and self-efficacy were 3.0, 3.3 [26], and 0.7 [37], respectively. In addition, the sample size was calculated according to the estimated effect size of 0.7 on self-efficacy, with a statistical power of 80% and ∂ = 0.5. Recruitment of 60 participants was intended to compensate for a 15% dropout during the intervention. Moreover, considering the referral rate of the hospital, recruiting 60 volunteers within half a year was feasible.

### Randomization and ethical approval

The study protocol was approved by the Medical Ethics Committee of the Xiangya Hospital, Central South University (approval number: 201906143) and was registered in Chinese Clinical Trial Registry (Registration Number: ChiCTR1900026550). Eligible participants were initially screened by a research assistant. All participants were asked to sign the informed consent. Subsequently, the participants were assessed by a blinded evaluator at baseline and were randomized. The random sequence was generated using a table of random numbers, and the randomization results were stored in a numbered, sealed, and opaque envelope by another research assistant.

### Statistical analysis

SPSS 22.0 was used for statistical analyses. Descriptive statistics were conducted for all outcome variables. Before the analyses, Shapiro–Wilk test was performed to test the normality of continuous data. Independent t-test and Mann Whitney U test were employed to compare normally distributed data and non-normally distributed data between the two groups, respectively. Wilcoxon signed-rank test was performed to compare within-group differences. Chi-square test or Fisher's exact test was used to compare categorical data. Rank sum test was used to compare ordinal variables. Minimal clinically important differences (MCID) were applied to compare the present results. Statistical significance was set at *P* value < 0.05. Intent-to-treat analysis [38] was applied in in all comparisons, with Last Observation Carried Forward Method for the imputation of any missing value.

## Results

### Participants and recruitment

**Fig. 1:**
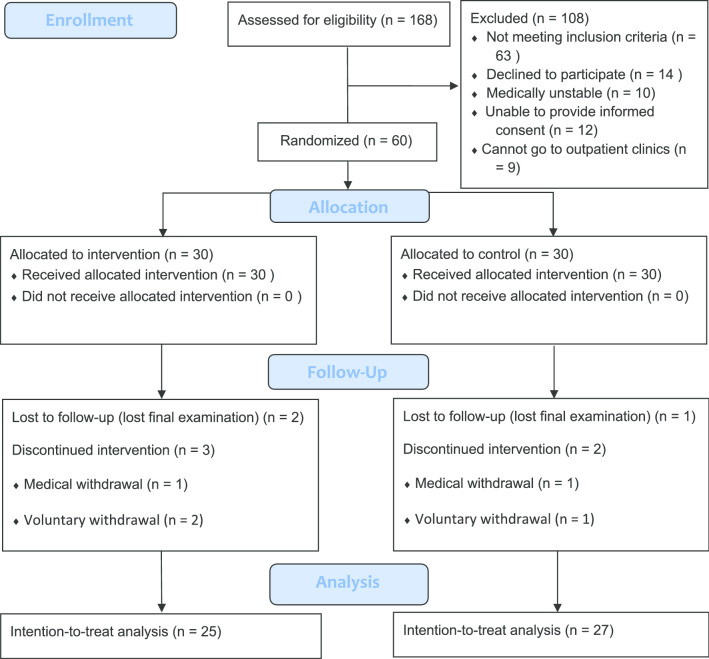
Flow chart of study

A total of 168 participants were identified in this study (Fig. 1). Demographic characteristics were presented and homogeneity of the groups at baseline was confirmed in Table 1. The overall attrition rate was 13.3%. No falls or other adverse reactions were reported.

**Table 1:** Characteristics of included participants

	VR group (n = 25)	Control group (n = 27)	*P* value
Age (years)*	53.28 ± 15.30	54.11 ± 14.81	0.430
Post-stroke duration (days)†	20 (9–45.5)	8 (6–15)	0.125
Mini-mental state evaluation†	28 (24.5–29)	28 (26–29)	0.918
Intervention duration†	8 (6–14.5)	7 (6–10)	0.610
Gender			0.335
Male	18 (72)	16 (59)	
Female	7 (28)	11 (41)	
Etiology			0.244
Ischemic	21 (84)	19 (70)	
Hemorrhage	4 (16)	8 (30)	
Paretic side			0.099
Right	12 (48)	7 (26)	
Left	13 (52)	20 (74)	
Handedness			1.000
Right	25 (100)	26 (96.3)	
Left	0 (0)	1 (3.7)	
Muscle strength (shoulder flexion)			0.635
2	3 (12)	6 (22.2)	
3	12 (48)	11 (40.7)	
4	6 (24)	5 (18.5)	
5	4 (16)	5 (18.5)	
Muscle tone (elbow flexion)			0.812
0	21 (84)	22 (81.5)	
1	4 (16)	5 (18.5)	
Educational level			0.372
Primary school	6 (24.0)	9 (33.3)	
Middle school	5 (20.0)	7 (25.9)	
High school	4 (16.0)	2 (7.4)	
Associate’s degree	5 (20.0)	5 (18.5)	
Bachelor’s degree or above	5 (20.0)	4 (14.8)	

Values are numbers (percentages) unless otherwise stated

^*^Means ± standard deviation

^†^Median (interquartile range)

### Primary outcome

A significant between-group difference in SSEQ was found after intervention (Median Difference = 8, *P* = 0.043, Z = − 2.027), and only daily activities domain in SSEQ demonstrated significance (Median Difference = 6, *P* = 0.017, Z = − 2.392). No significant differences in COPM were found between two groups. Table 2 shows the details of the main activities of the participants. The rate of difficult activities in the areas of self-maintenance, productivity, and leisure was 48.2%, 25.8%, and 25.8%, respectively. The median and interquartile range of the results were shown in Table 3.

**Table 2:** Main activities reported by the participants in the COPM domain (n = 52)

COPM domain	COPM category	Activity	Absolute frequency
Self-maintenance (75, 48.4%)	Personal care (33, 21.3%)	Eating	13
		Grooming	5
		Dressing	8
		Taking a bath	7
	Functional mobility (27, 17.4%)	Transfer	6
		Walking	21
	Independence away from home (15,9.7%)	Using public transport	4
		Driving	7
		Shopping	4
Productivity (40, 25.8%)	Work (18, 11.6%)	Work activities	18
	Domestic tasks (21, 13.5%)	Making a meal	4
		Wash clothes	4
		Caring children	6
		Housework	7
	School (1, 0.6%)	Learning	1
Leisure (40, 25.8%)	Quiet recreation (20, 12.9%)	Watching TV	4
		Playing chess	1
		Playing game	1
		Playing phone	2
		Reading	2
		Playing mahjong	10
		Writing	2
	Active recreation (11, 7.1%)	Traveling	2
		Playing ball (basketball, ping-pong)	3
		Fishing	1
		Climbing mountain	1
		Swimming	2
		Playing erhu	1
		Running	1
	Socialization (9, 5.8%)	Join a party	6
		Visiting	3

**Table 3:** Outcome measure scores (median and IQR) at baseline (T1) and 3-week follow-up (T2)

Outcome	VR group (n = 25)			Control group (n = 27)			Comparison Between group (*P*^b^)	
	T1	T2	*P*^a^	T1	T2	*P*^a^	T1	T2
MBI	84 (66.5–95)	95 (88.5–100)	< 0.000^*^, Z = – 3.922	65 (52–86)	85 (59–100)	< 0.000^*^, Z = – 4.202	0.081, Z = − 1.742	0.030^*^, Z = − 2.171
FMU	53 (40–59.5)	62 49–64.5)	< 0.000^*^, Z = – 4.296	45 (23–59)	58 (31–64)	< 0.000, Z = – 4.546	0.244 Z = − 1.164	0.295 Z = − 1.047
FTHUE	5 (3–7)	7 (4–7)	0.001^*^, Z = – 3.375	4 (3–6)	6 (4–7)	< 0.000^*^, Z = – 4.167	0.237 Z = − 1.182	0.191 Z = − 1.307
COPM								
Performance	5 (3–6)	7.3 (5–8.5)	0.002^*^, Z = – 3.173	4 (2.25–5.75)	7 (5–8.4)	< 0.000^*^, Z = – 3.836	0.414 Z = − 0.816	0.607 Z = − 0.514
Satisfaction	5 (3–6.5)	7.3 (5–8.7)	0.001^*^, Z = – 3.232	4.5 (2.67–7.5)	7 (5.5–9.2)	0.001^*^, Z = – 3.271	0.985 Z = − 0.018	0.920 Z = − 0.101
SSEQ								
Total	117 (96.5–125.5)	125 (109.5 –130)	0.006^*^, Z = – 2.744	108 (91–122)	117 (102–125)	0.031^*^, Z = – 2.155	0.305 Z = − 1.026	0.043^*^ Z = − 2.027
Daily activities	72 (55.5–77.5)	78 (66.5–80)	0.006^*^, Z = – 2.767	68 (51–74)	72 (60–76)	0.096, Z = – 1.665	0.192 Z = − 1.304	0.017^*^ Z = − 2.392
Self-management	45 (38.5–47)	49 (40.5–50)	0.073, Z = – 1.792	43 (38–48)	46 (40–50)	0.009^*^, Z = – 2.598	0.659 Z = − 0.441	0.571 Z = − 0.566

All values are Median (IQR, interquartile range). n number of patients;

*VR* virtual reality, *MBI* modified Barthel Index, *FMU* Fugl-Meyer assessment-upper extremity, *FTHUE* functional test for the hemiplegic upper extremity, *COPM* canadian occupational performance measure, *SSEQ* stroke self-efficacy questionnaire

^a^The Wilcoxon signed-rank test was used to compare within-group differences

^b^The Mann Whitney U-test was used to compare between-group differences

*P ≤ .05

### Secondary outcome

A significant difference in MBI was found between groups (Median Difference = 10, *P* = 0.03, Z = − 2.171; Table 3). No significant between-group difference in FMA-UE and FTHUE was noted, and both groups had improved upper limb function (*P* < 0.05). The results of FMA in both groups were more than 7.25 points (MCID) and showed clinical significance.

## Discussion

In this study, we found that additional 3-week VR training could improve self-efficacy and ADL; however, the occupational performance, satisfaction, and upper extremity function were not superior in participants who had VR training.

About half of the demands of COPM were in the area of self-maintenance, such as personal care and functional mobility; this finding was similar to that of another study [39]. Specifically, walking was the most frequently mentioned activity of patients with stroke, which was followed by eating and dressing. Moreover, the most important activity in the area of productivity was working, which was followed by housework; this finding was also similar to previous research results [40, 41]. For leisure, the demands appeared to be of diversity and be related to patients’ interests. Most patients considered playing mahjong as the first priority for leisure activities, and this might be associated with the Chinese culture. Besides, social engagement is associated with the interaction of different occupations, which can support expected community and family involvement, as well as activities involving peers and friends [42].

In our study, positive changes in the score of occupational performance were observed in both groups; the score was 2 points greater than the baseline, indicating a clinically significant improvement in occupational performance, and no between-group difference was found. The result of our study was consistent with those of current studies [26, 43], although the number of VR-related studies using occupational performance as an outcome indicator is insufficient. Furthermore, it was reported that a patient-centered treatment enables therapists to fully consider each patient's satisfaction, desire, and goals, which in turn makes the promotion of enthusiasm among participants easier, thereby resulting in adherence to intervention and patient satisfaction [26]. There are some differences between the VR therapeutic activities and realistic daily activities. These barriers were very common in the implementation of VR training, which need the collaboration of engineers and game designers, clinicians and end-users to improve them [44]. In addition, the demand of productive activities and recreational activities was about 52%, and target training in our VR training program was insufficient. Moreover, because of the relatively large sample size and short training protocol compared with two previous randomized controlled trials [31, 43], implementing a patient-centered treatment in clinical practice was difficult. These results may explain the absence of a significant difference between two groups in COPM. Nevertheless, the inherent interactivity and entertainment of VR can motivate patients’ participation.

Bandura’s cognition theory was commonly applied for stroke self-management program, and this kind of program contained meaningful and physical activities to further improve self-efficacy [45–47]. Similar to stroke self-management program, VR can provide task-oriented, imitative, and repetitive activities, and it also has the advantage to integrate these four principles. Additionally, according to theoretical concepts of Bandura’s self-efficacy, the most feasible way to assist participants to obtain and improve self-efficacy is to use self-efficacy-related four principles [6, 48]. The human-shaped model displayed on the screen can help participants obtain indirect experiences, and participants could acquire successful experience through repetitive activities. Meanwhile, appropriate concern and encouragement by therapists and families can be provided for participants during the training. Hence, participants can improve self-efficacy by integrating Bandura’s principles into VR training program.

Currently, studies that used holistic self-efficacy as an outcome indicator in VR training are insufficient, and only a few studies investigated balance self-efficacy [22, 33, 49, 50]. Previous studies found that VR could increase participants' confidence, motivation to participate, and functional activities based on the remodeling and reorganization of brain function [26, 51, 52]. Observational learning through VR training can activate mirror neurons in cortex. Similarly, participants receiving sensory feedback in VR training were likely to learn the target motor behavior. The feedback could promote the development of use-dependent cortical plasticity, leading to the recovery of motor function [53]. Moreover, the functional improvement induced by motor learning through VR training could greatly improve participants' confidence and self-efficacy in a new environment [22, 32, 33, 49, 50]. In addition, a study reported that participants regarded VR training as an interactive and enjoyable sports game rather than a training; thus, they were more motivated to engage in the training [54]. Moreover, another advantage of VR is that it can greatly save labor.

Our study found that VR training is beneficial for patients with stroke in terms of ADL but not upper limb function. These results were consistent with those of a previous study [21]. The possible reasons may be as follows: the overall intervention time of this study was relatively short. Although a review confirmed that a total intervention time more than 15 h would become a trend of customization [21], the regulations of medical insurance prevented us from having a longer treatment duration. Additionally, the VR equipment used in this study was mainly focused on the proximal joint of the upper limb, and thus lacked functional training for the distal joints (hand and wrist), which possibly contributed to the difficulty in enhancing upper limb function scores. In our study, both self-efficacy and ADL improved compared with control group. The improvement of ADL might be related to the improvement of self-efficacy. A previous study revealed that patients with high self-efficacy performed better in daily activities than patients with low self-efficacy [4].

### Limitations

This study has some limitations. Sample size was small in this study. Although the intervention protocol was designed completely, it did not exactly match the daily activities. Moreover, to achieve blinding and consistency, the evaluation was performed by the same research therapist who had no clinical relationship with the participant. However, subjective evaluations commonly require a certain degree of interactivity to better complete, such as COPM and self-efficacy evaluation. This study did not have any follow-up assessments to track continuous effects. All these limitations need to be reconsidered in future studies.

## Conclusions

According to the results of this study, VR training could be used as an effective tool to enhance self-efficacy and improve ADL of patients with stroke in acute hospitals. Our study used self-efficacy, occupational performance and satisfaction as primary outcomes, and thus added important outcomes to VR researches. Nonetheless, more randomized controlled trials are needed to determine the effectiveness of VR training on occupational performance and satisfaction of patients with stroke.

## Data Availability

Raw data is available from the corresponding author on reasonable request.

## Abbreviations

VR: Virtual reality
COPM: Canadian occupational performance measure
SSEQ: Stroke self-efficacy questionnaire
MBI: Modified Barthel Index
FMA-UE: Fugl-Meyer assessment-upper extremity
FTHUE: Functional test for the hemiplegic upper extremity
ADL: Activities of daily living
